# Supplementary material for: State-dependent binding of cholesterol and an anionic lipid to the muscle-type Torpedo nicotinic acetylcholine receptor
Source: Commun Biol. 2024 Apr 10;7:437. doi: 10.1038/s42003-024-06106-8 (PMC11006840; doi:10.1038/s42003-024-06106-8)
Supplement: Supplementary file 2 — Supplementary Information [file 42003_2024_6106_MOESM2_ESM.pdf]

Supplemental Information

State-dependent binding of cholesterol and an anionic lipid to the muscle-type *Torpedo* nicotinic acetylcholine receptor

Anna Ananchenko[1], Ruiyan Gao[1], François Dehez*[2], John E. Baenziger*[1].

1. Department of Biochemistry, Microbiology and Immunology, University of Ottawa, Ottawa, Canada
2. Laboratoire de Physique et Chimie Théoriques, Université de Lorraine, Nancy, France

*To whom correspondence should be addressed: John E. Baenziger, Department of Biochemistry, Microbiology, and Immunology, University of Ottawa, 451 Smyth Rd. Ottawa, ON, K1H 8M5, Canada, Tel.: (613) 562-5800 x8222; Fax.: (613) 562-5440; E-mail: John.Baenziger@uottawa.ca; Francois Dehez, C LPCT, UMR 7019 Université de Lorraine CNRS, Vandœuvre-lès-Nancy, F-54500, France, Tel.: +33-(0)3-83-68-40-98 ; Fax.: +33-(0)3-83-68-43-87 ; E-mail.: Francois.Dehez@edam.uhp-nancy.fr

Table S1. CG simulations system setup

System	Lipids	No. of repeats	Box Size	No. of Atoms	No. of Water Molecules	Salt Concentration
Nicotine-bound state, Pure PC	947 POPC	3	180 x 180 x 200 Å	55355	38515	0.15 mM
Apo state, Pure PC	942 POPC	3	180 x 180 x 200 Å	55290	38493	0.15 mM
Nicotine-bound state, 3:2 PC:PA	378 POPA<br>569 POPC	3	180 x 180 x 200 Å	55374	38172	0.15 mM
Apo state, 3:2 PC:PA	377 POPA<br>566 POPC	3	180 x 180 x 200 Å	55339	38169	0.15 mM
Nicotine-bound state, 3:2 PC:Chol	378 CHOL<br>568 POPC	3	180 x 180 x 200 Å	54218	38906	0.15 mM
Apo state, 3:2 PC:Chol	377 CHOL<br>566 POPC	3	180 x 180 x 200 Å	54195	38910	0.15 mM
Nicotine-bound state, 3:1:1 PC:PA:Chol	189 CHOL<br>189 POPA<br>568 POPC	3	180 x 180 x 200 Å	54790	38533	0.15 mM
Apo state, 3:1:1 PC:PA:Chol	188 CHOL<br>188 POPA<br>566 POPC	3	180 x 180 x 200 Å	54771	38552	0.15 mM
Nicotine-bound state, 3:2 PC:monoanionic PA	378 mono-POPA<br>568 POPC	3	180 x 180 x 200 Å	55380	38556	0.15 mM
Apo state, 3:2 PC:monoanionic PA	377 mono-POPA<br>566 POPC	3	180 x 180 x 200 Å	55345	38550	0.15 mM

Table S2. Atomistic simulations system setup

System	Lipids	No. of repeats	Box Size	No. of Atoms	No. of Water Molecules	Salt Concentration
Nicotine-bound state, Pure PC	367 POPC	2	130 x 130 x 190 Å	315132	78192	0.15 mM
Apo state, Pure PC	378 POPC	2	130 x 130 x 190 Å	316587	78148	0.15 mM
Nicotine-bound state, 3:2 PC:PA	150 POPA<br>221 POPC	2	130 x 130 x 190 Å	316485	78836	0.15 mM
Apo state, 3:2 PC:PA	160 POPA<br>218 POPC	2	130 x 130 x 190 Å	317123	78756	0.15 mM
Nicotine-bound state, 3:2 PC:Chol	247 CHOL<br>312 POPC	2	130 x 130 x 190 Å	325203	77460	0.15 mM
Apo state, 3:2 PC:Chol	239 CHOL<br>317 POPC	2	130 x 130 x 190 Å	325030	77324	0.15 mM
Nicotine-bound state, 3:1:1 PC:PA:Chol	94 POPA<br>101 CHOL<br>266 POPC	2	130 x 130 x 190 Å	320966	78044	0.15 mM
Apo state, 3:1:1 PC:PA:Chol	93 POPA<br>102 CHOL<br>261 POPC	2	130 x 130 x 190 Å	321640	78476	0.15 mM

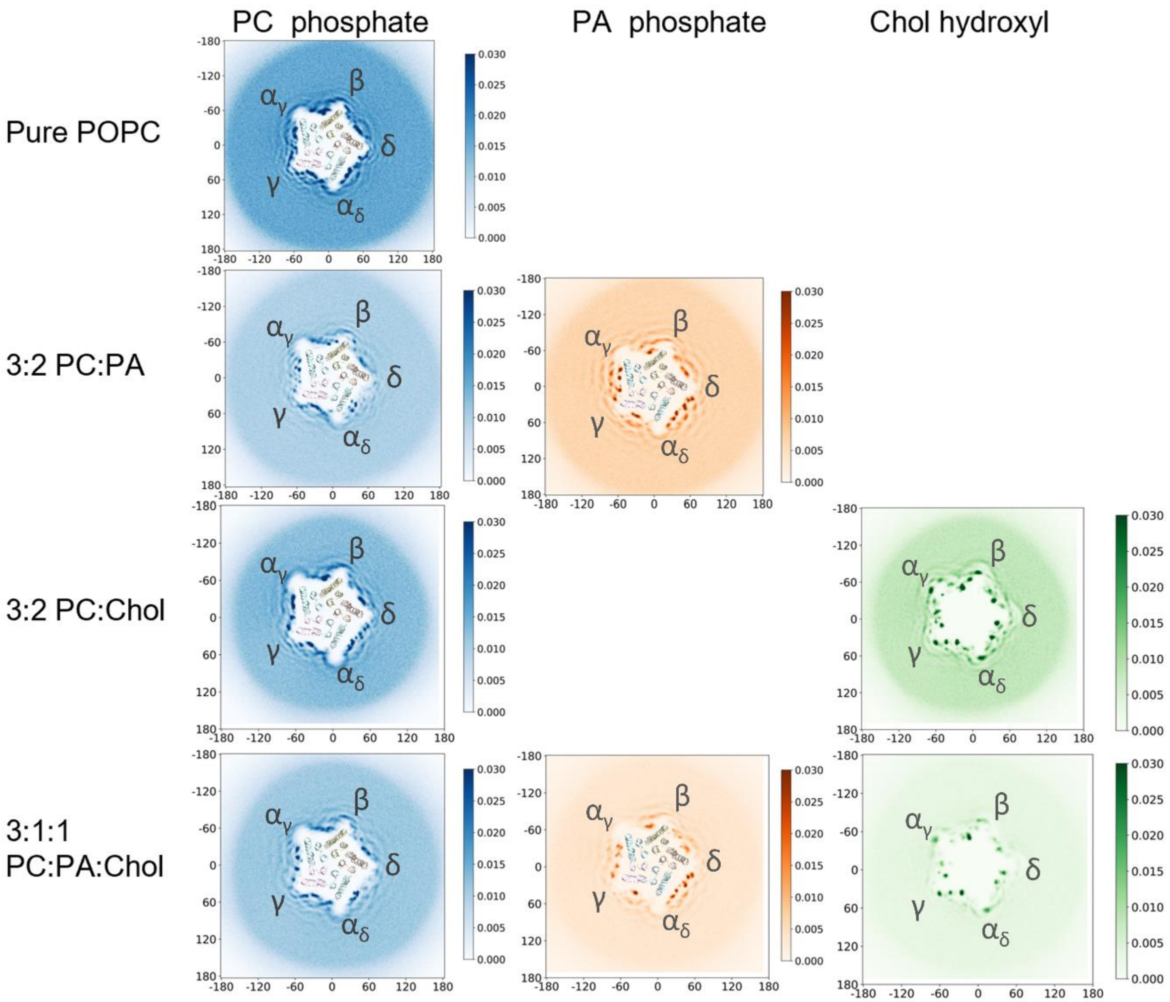

Figure S1. Top-down 2D density plots for lipid headgroups localizing around the apo nAChR in the outer leaflet. Density calculated over the course of 3 x 30μs CG-MD simulations. Density is based on the "PO4" bead for both PA and PC, and the "ROH" bead for cholesterol. Top-down subunit arrangement is shown.

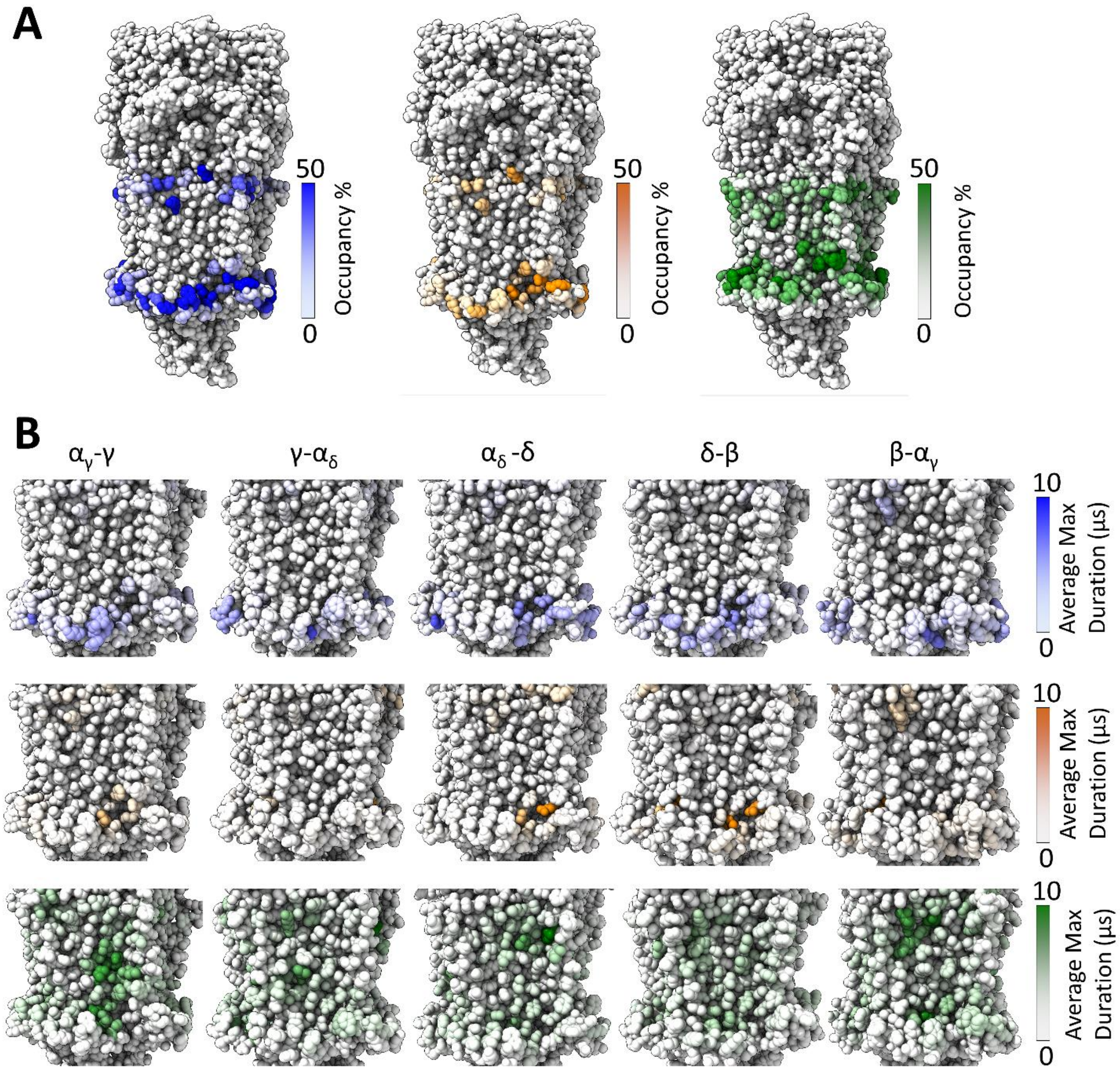

Figure S2. Occupancy and average maximum duration profiles for lipids interacting with the apo state. A) Occupancy values (% of frames with an interacting lipid) for residues interacting with the headgroups of PC, PA or Chol (left to right blue, orange, green) are mapped onto the atomistic apo nAChR structure (PDB: 7QKO). In each case, a side view of the α_γ-γ subunit interface is shown. **B)** The average maximum duration for lipid headgroup binding (the average of the single longest interaction in each of three CG-MD simulation repeats of 30 µs) mapped onto the atomistic apo nAChR structure at each subunit interface. Top, PC (blue); middle, PA (orange); bottom, Chol (green).

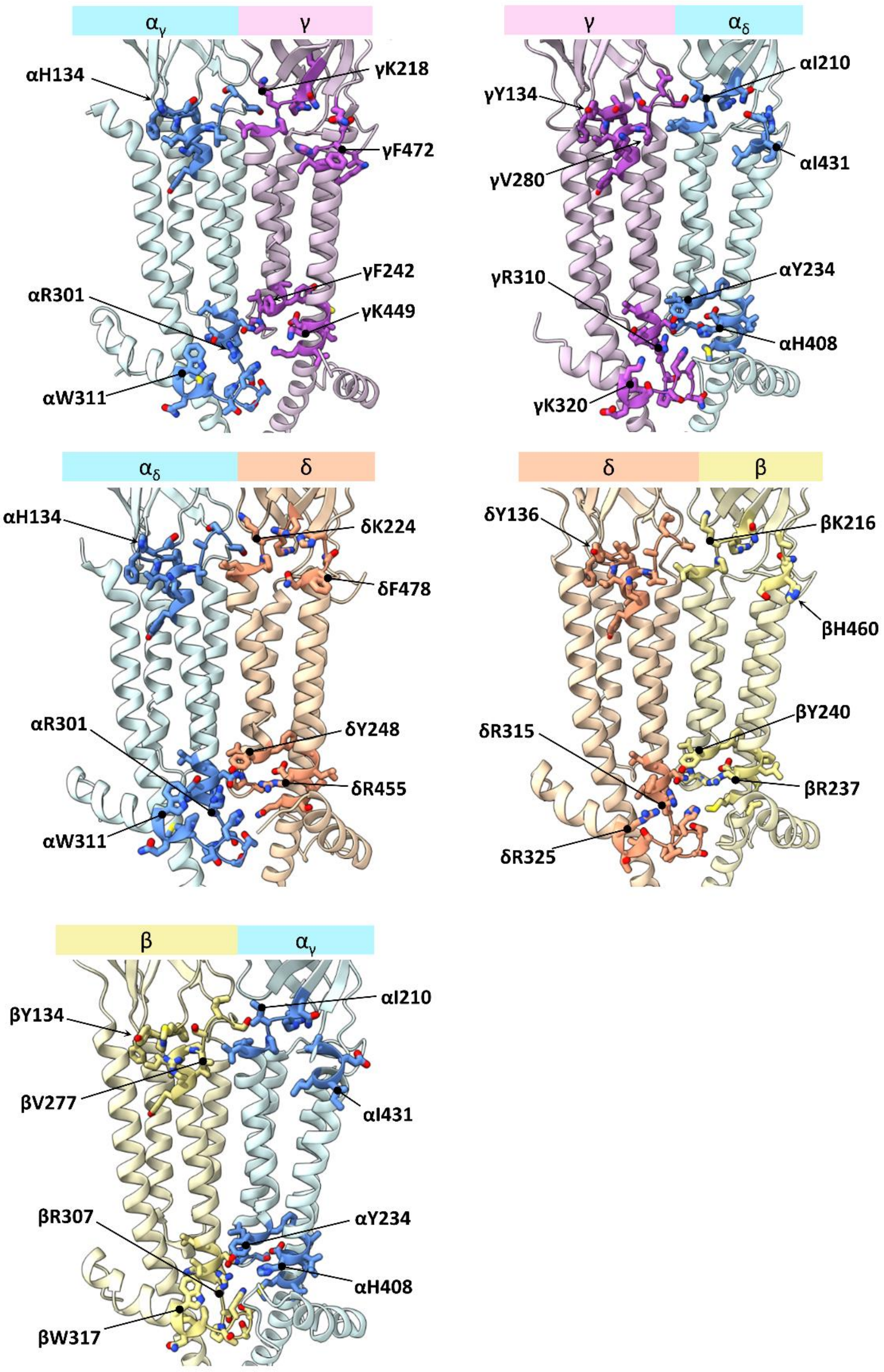

Figure S3. Residues that exhibit high-occupancy interactions with lipid headgroups in the apo state. High occupancy interacting residues are shown as sticks, with key residues labelled (see Figs. S4, S6-S8). Subunit backbones are shown as cartoons.

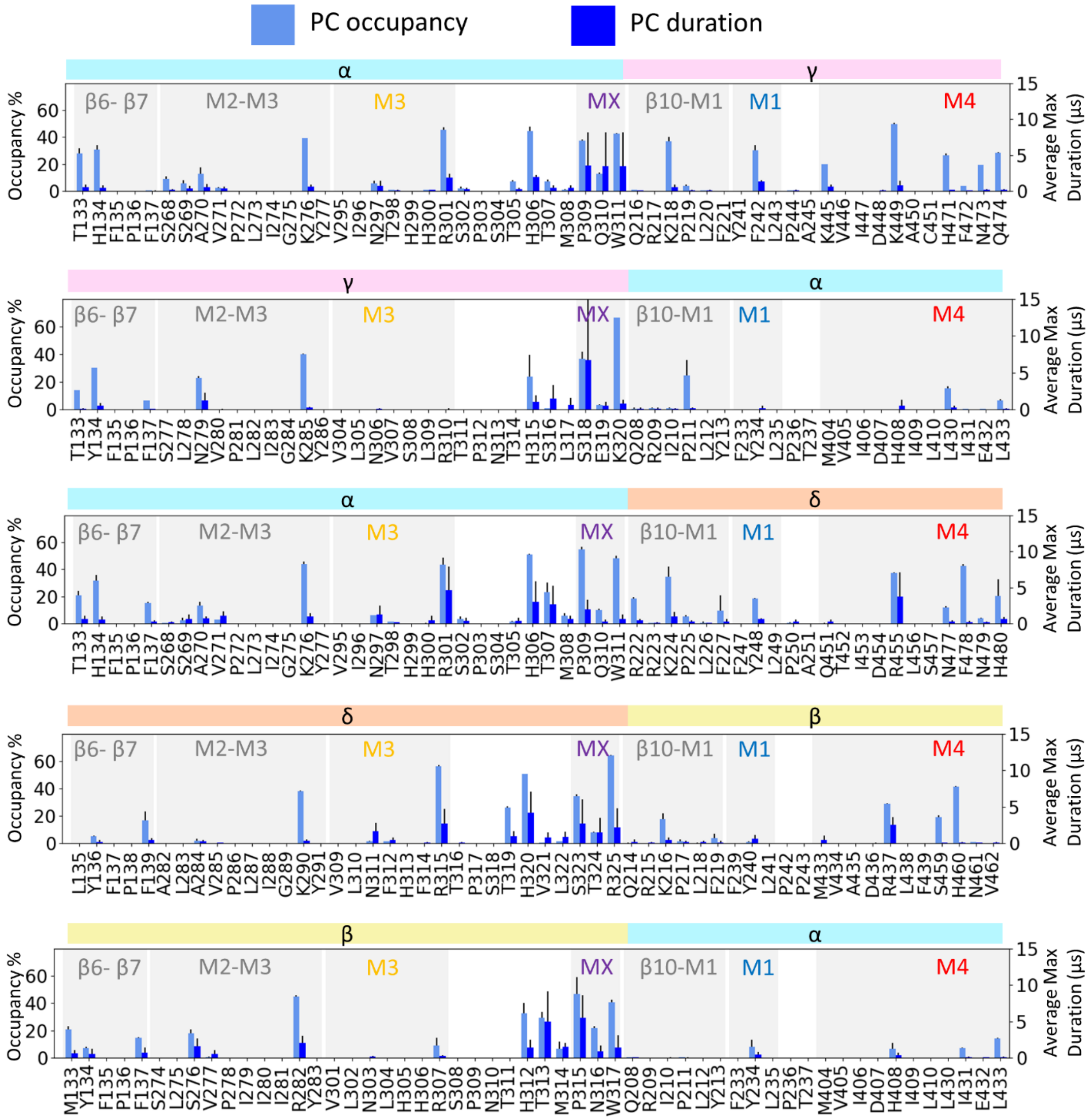

Figure S4. Dual axis occupancy/average maximum durations plots for PC headgroup (PO_4 bead) binding to the apo-nAChR imbedded in a pure PC membrane. Data taken from 3 x 30 μs CG-MD simulation. Error bars represent standard deviations. Structures corresponding to the lipid-interacting regions are shown in Fig. S3.

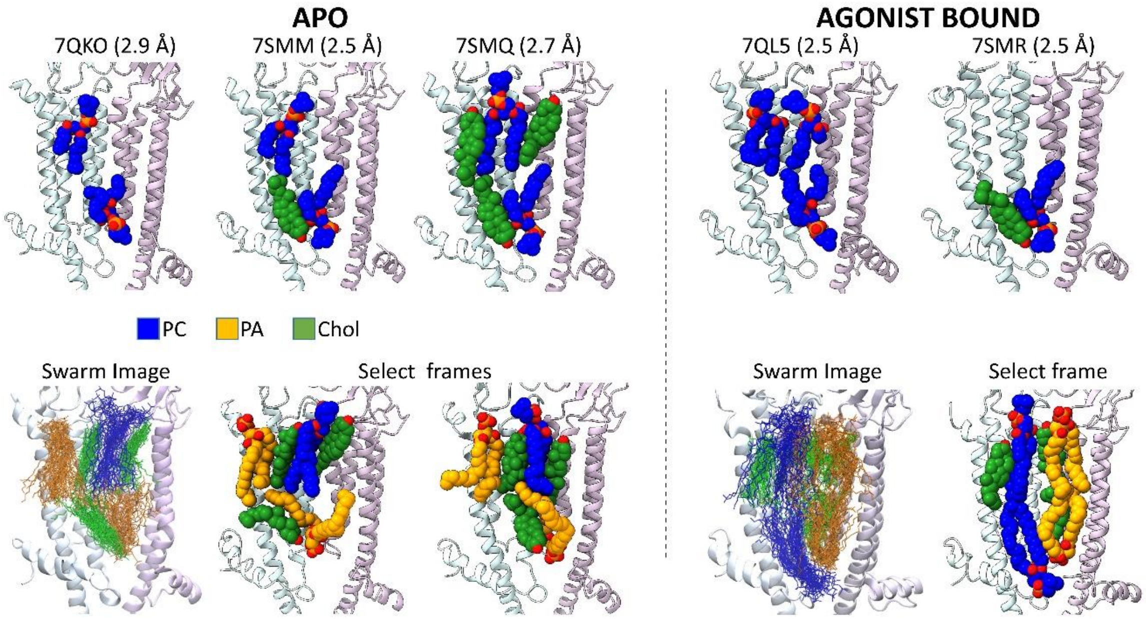

Figure S5. Comparison of the lipid binding observed in the cryo-EM structures and the MD simulations. The top row shows the α_γ-γ interface of *Torpedo* nAChR structures with modelled lipids in apo and agonist-bound states. Note that for 7QKO and 7Ql5, the nAChR was purified in the *presence* of exogenous soybean azolectin, which facilitates exchange with endogenous lipids, such as Chol, and then reconstituted into azolectin MSP2N2 nanodiscs. For 7SMM, 7SMQ, and 7SMR, the nAChR was purified in the *absence* of exogenous lipid and thus retains endogenous Chol. Exogenous Chol was also added to the purified nAChR that yielded the 7SMQ structure. For these structures, the nAChR was then reconstituted into either azolectin or azolectin plus Chol (7SMQ) saposin nanodiscs. Protein subunits are shown as light cyan (α_γ) and light purple (γ) cartoons, and bound lipids are shown as spheres. The botton row shows lipid binding poses observed in atomistic simulations of the apo (7QKO) (left) and nicotine-bound (7Ql5) (right) states imbedded in 3:1:1 PC:PA:Chol membranes. In each case, a *select* representative frame from the CG-MD simulations was back-mapped to a full atom structure and then atomistic simulations run for 250 ns. The left image for both the apo and nicotine-bound structures is a swarm image, where the pose of each lipid at 8 ns intervals over the course of the 250 ns simulation is shown as sticks – the resulting 31 poses for each lipid are superimposed on top of each other. Frames were aligned to the C_α atoms of the α_γ and γ subunits. The adjacent images to the right are select frames where the bound lipids are shown as spheres. The selected frames also illustrate the variety and dynamics of lipid binding observed in the MD simulations, with some frames matching closely the lipid binding poses observed in cryo-EM structures and others capturing other distinct binding poses. For example, the two frames from the atomistic simulation for the apo state reveal lipid binding poses that match closely those observed in the apo+Chol cryo-EM structure, with some of the same poses captured in the other apo structures. The selected frame for the nicotine-bound state reveal additional lipid binding poses as discussed in the text, such as the binding of PC with its headgroup projecting between the two MX α-helices from the principal and complementary subunits (in this frame the PC acyl chains outcompete Chol for binding) and Chol bound deeper to the high affinity inner leaflet PA binding site. The simulations also highlight sites where PA outcompetes PC for binding, particularly to inner leaflet sites. PC, blue; PA, orange; Chol, green.

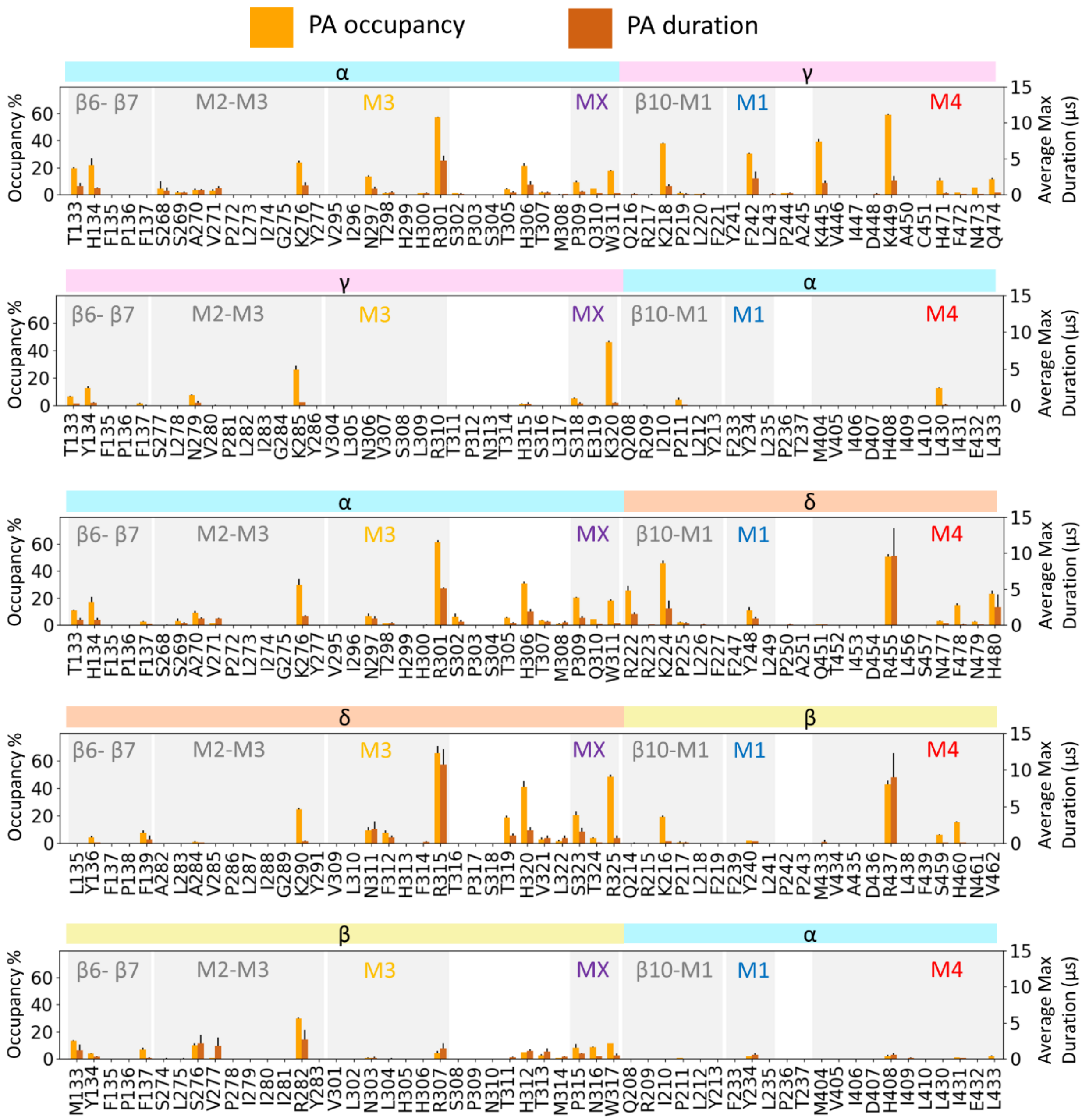

Figure S6. Dual axis occupancy/average maximum duration plots for PA headgroup (PO_4 bead) interactions with prominent residues of the apo nAChR imbedded in a 3:2 PC:PA membrane. Data taken from 3 x 30 µs CG-MD simulation. Error bars represent standard deviations. Structures corresponding to the lipid-interacting regions are shown in Fig. S3.

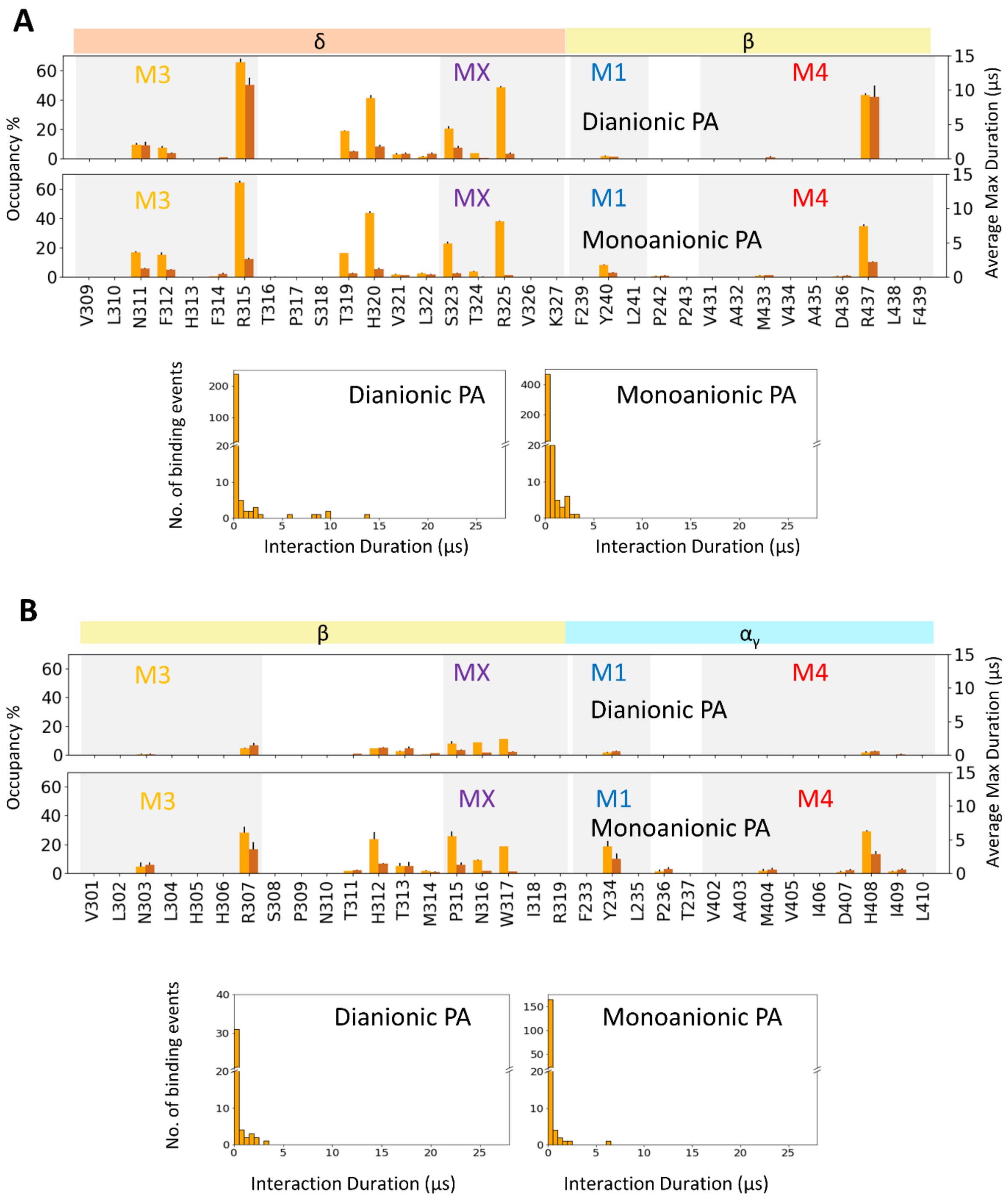

Figure S7. Interactions of monoanionic versus dianionic PA with the apo nAChR in a 3:2 PC:PA membrane. A) The top two panels are dual-axis occupancy/duration plots for monoanionic and dianionic PA binding to residues near the inner leaflet binding site at the δ-β subunit interface. Error bars represent standard deviations. The bottom panel shows interaction duration histograms for both monoanionic and dianionic lipid headgroup interactions with δR315, showing all interactions from 3 x 30 μs CG-MD simulations. **B)** Same data as in (A) but for the β-α inner leaflet binding site. Structures corresponding to the lipid-interacting regions are shown in Fig. S3.

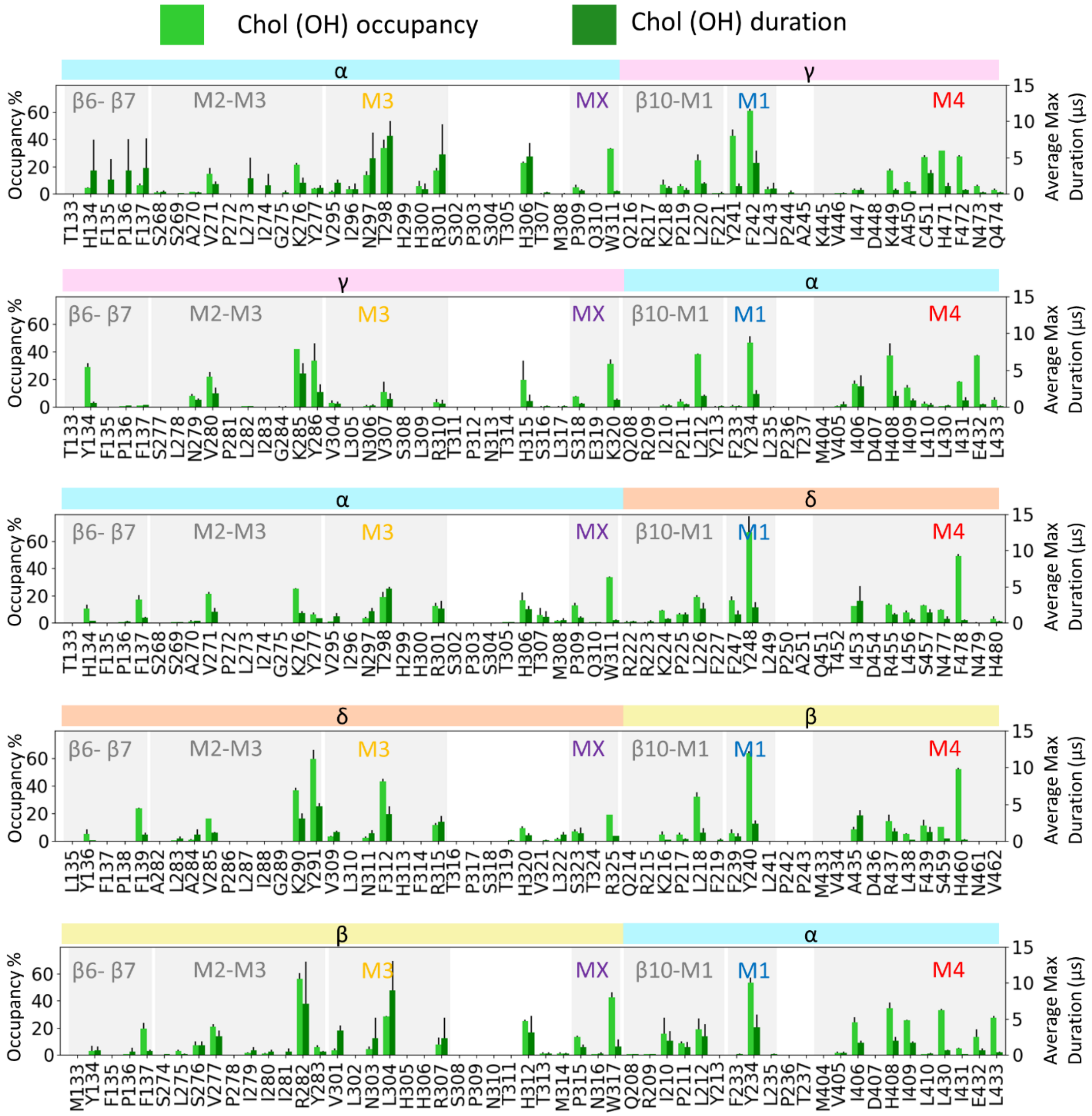

Figure S8. Dual axis occupancy/average maximum duration plots for Chol (ROH) interactions with prominent residues in a 3:2 PC:Chol membrane. Data taken from 3 x 30 µs CG-MD simulation. Error bars represent standard deviations. Structures corresponding to the lipid-interacting regions are shown in Fig. S3.

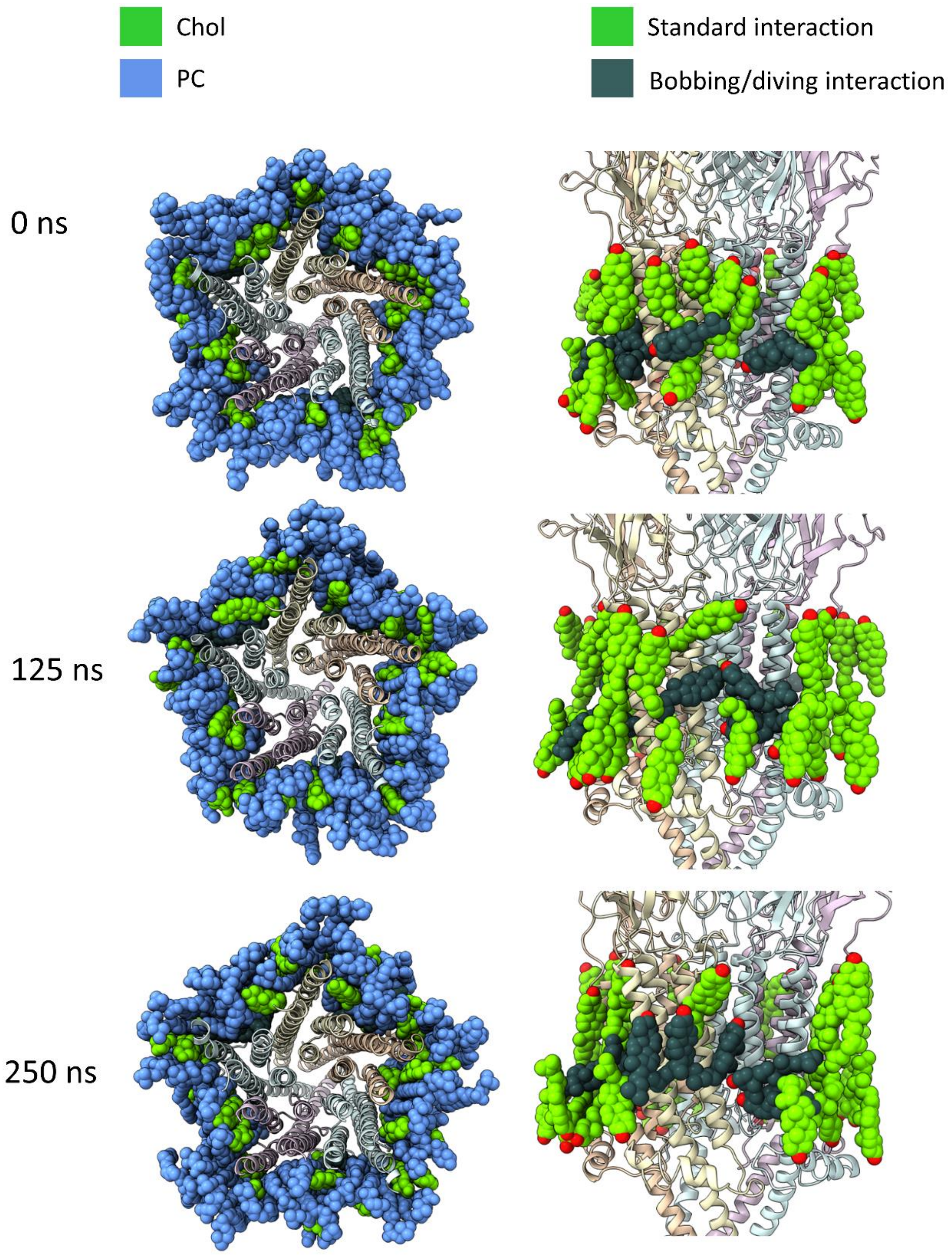

Figure S9. Snapshots of PC and Chol binding to the apo nAChR in a 3:2 PC:Chol membrane. Images show both PC and Chol binding at 0, 125 and 250 ns corresponding to the beginning, middle and end of one atomistic simulation repeat. The left column is a top-down view of bound PC and Chol (lipids within 5 Å of the nAChR) with the lipids shown as spheres and the TMD shown as cartoon with subunits colored as in Fig. 1. The right column presents a side view of Chol interactions with the TMD. Chol bound to the canonical upper and lower leaflets is shown in light green spheres while Chol diving and bobbing into the membrane is shown as dark green spheres. In all cases, the hydroxyl is in red.

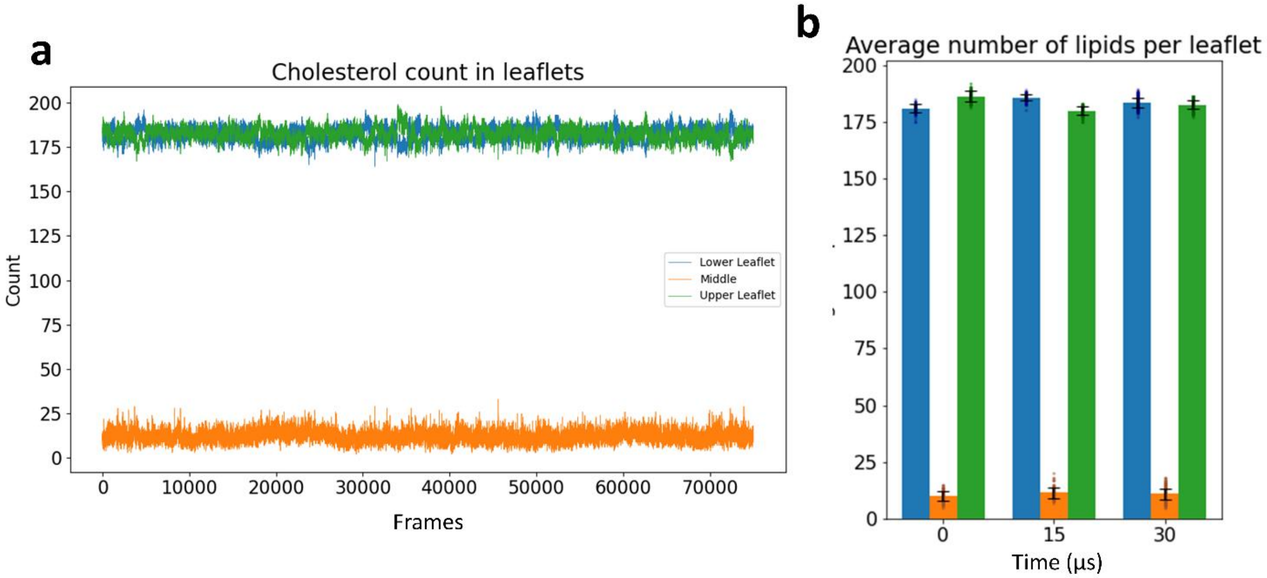

Figure S10. Cholesterol leaflet distribution over time. **A)** Number of Chol molecules at each simulation frame in a representative 30 µs long trajectory located with their headgroups (ROH) located in the canonical outer and inner leaflets or in the middle of the bilayer. Leaflets were defined as per LiPyPhilic method[72], with a midplane cutoff of 8 Å used to select Chol molecules in the middle of the bilayer. **B)** Number of Chol molecules in each leaflet or in the middle of the bilayer at the beginning, middle and end of the 30 µs simulation. The presented values are the mean for number of Chol molecules in each region over 100 frames, with error bars representing the standard deviation. Points represent Chol counts at each frame.

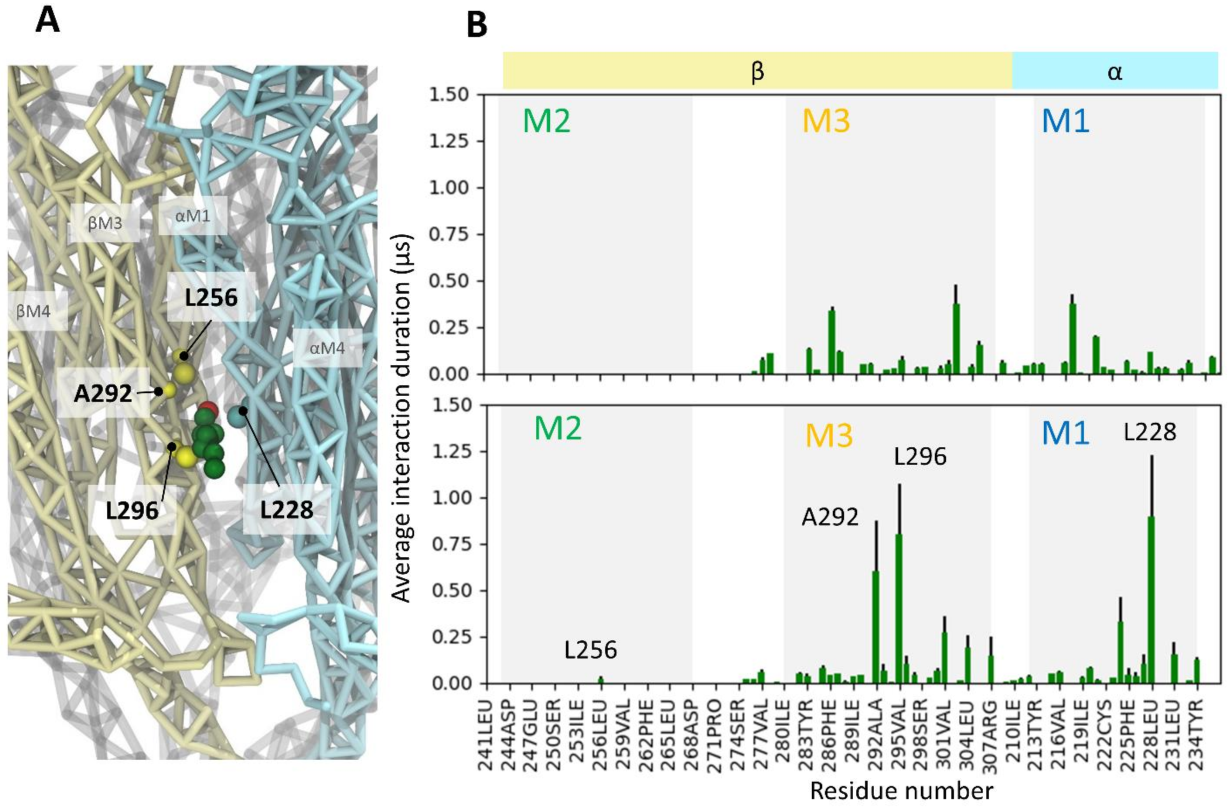

Figure S11. Chol interactions with the pore lining M2 helix in the nicotine-bound state. A) A single CG-MD simulation frame showing Chol interacting with Leu256 on the M2 α-helix of the β subunit. Protein backbone shown as sticks, with interacting residues and Chol shown as coarse-grained spheres. **B)** Average durations for Chol (ROH) interactions with residues at the β-α subunit interface in the apo (top) and nicotine-bound (bottom) states. Average interaction durations taken from 3 x 30 μs CG-MD simulations of each state in a 3:1:1 PC:PA:CHOL membrane. Error bars represent standard deviations.

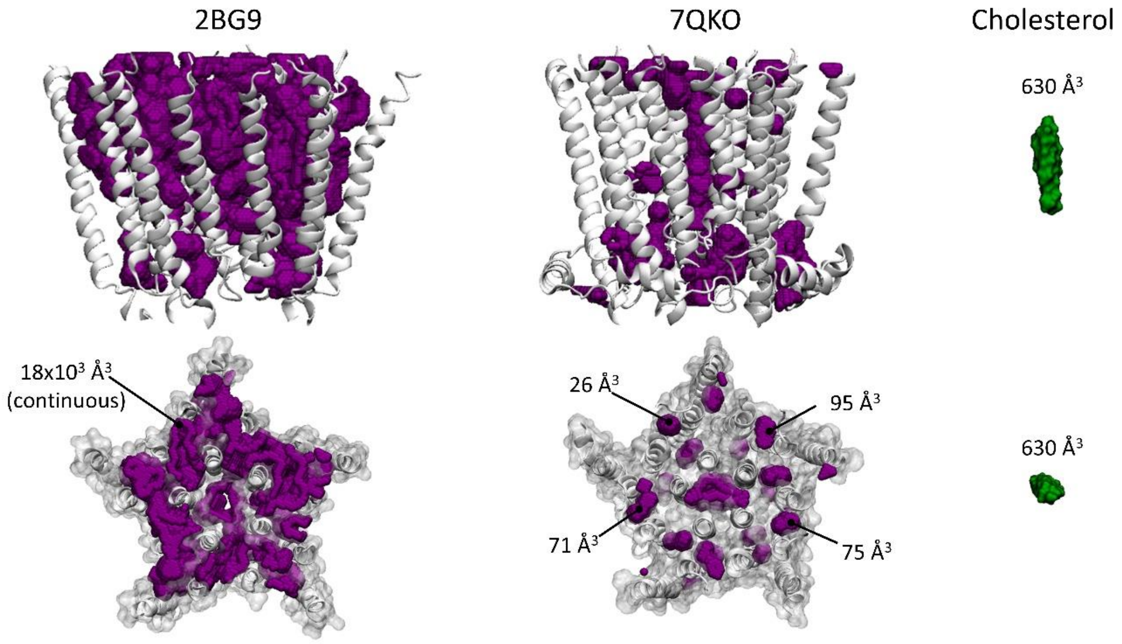

Figure S12. Comparison of the cavity volumes observed between TMD α-helices in the apo 2BG9 (4 Å resolution) and 7QKO (2.9 Å resolution) structures. Side view (top row) and top-down (bottom row) views of the cavities in 2BG9 are shown in purple on the left with the TMD α-helices shown as silver cartoons. Grey transparent surfaces are also shown in the top-down view (bottom row). **S**ide view (top row) and top-down views (bottom row) of the cavities in 7QKO with the same coloring as for 2BG9 are shown in the middle. A side view (top row) and a top-down view (bottom row) of Chol is shown on the right. Select upper leaflet cavity volumes are labelled. The cavities in the TMD of 2BG9 are continuous. Cavities were calculated using the KVFinder web tool[75]. The output of this tool is a series of points filling each cavity, which are shown here as spheres.

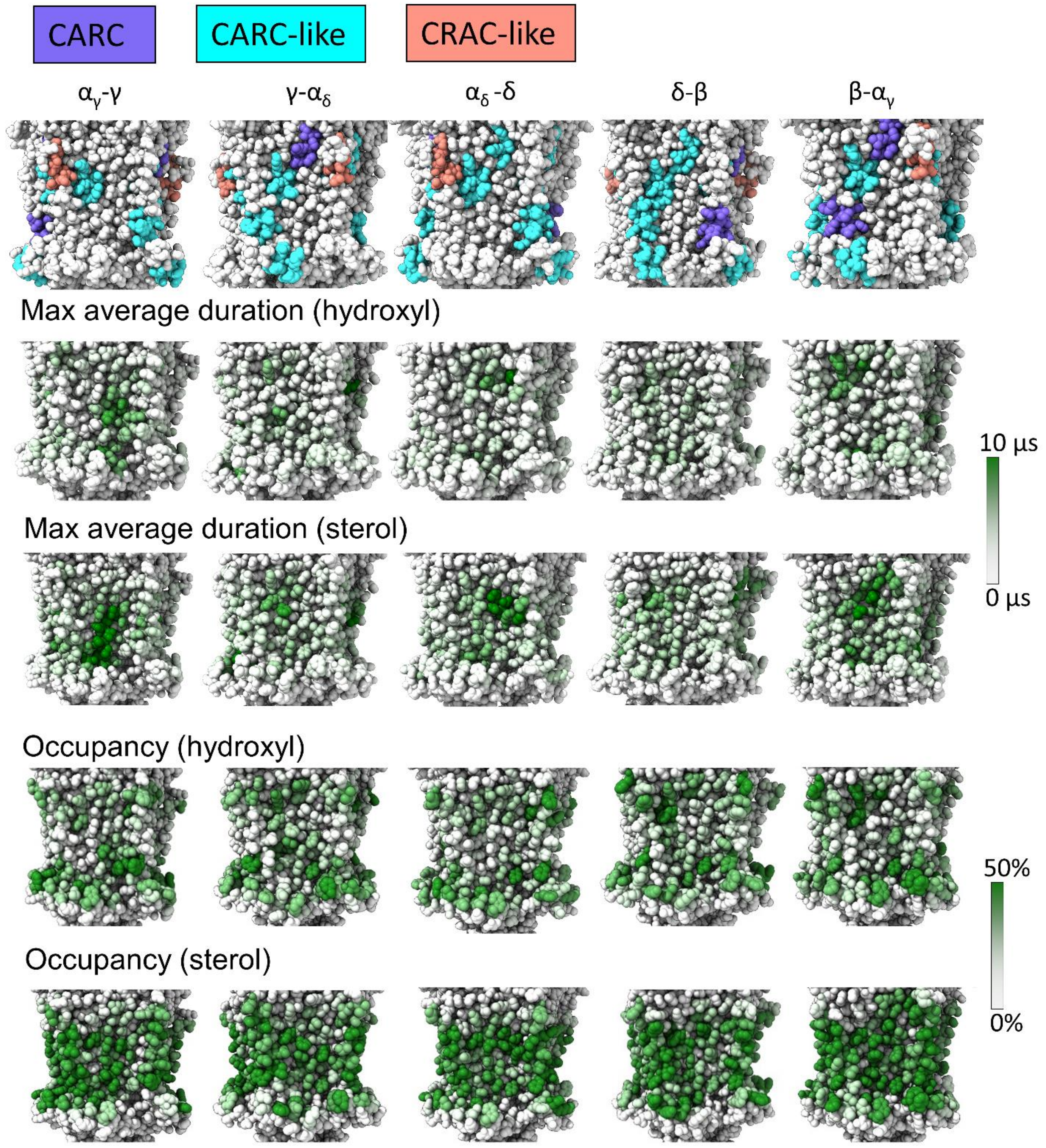

Figure S13. Cholesterol binding occurs in regions distinct from CRAC, CARC, and CARC-like motifs. The top row shows the location of CARC, CRAC-like and CARC-like motifs mapped onto each subunit-subunit interface in the TMD of the apo nAChR (PDB: 7QKO). The second and third rows show the average maximum duration of binding for the Chol hydroxyl and sterol groups, respectively. The fourth and fifth rows show the occupancy for the Chol hydroxyl and sterol groups, respectively. Data obtained from 3 x 30 µs CG simulation of the apo nAChR in a 3:2 PC:Chol membrane.

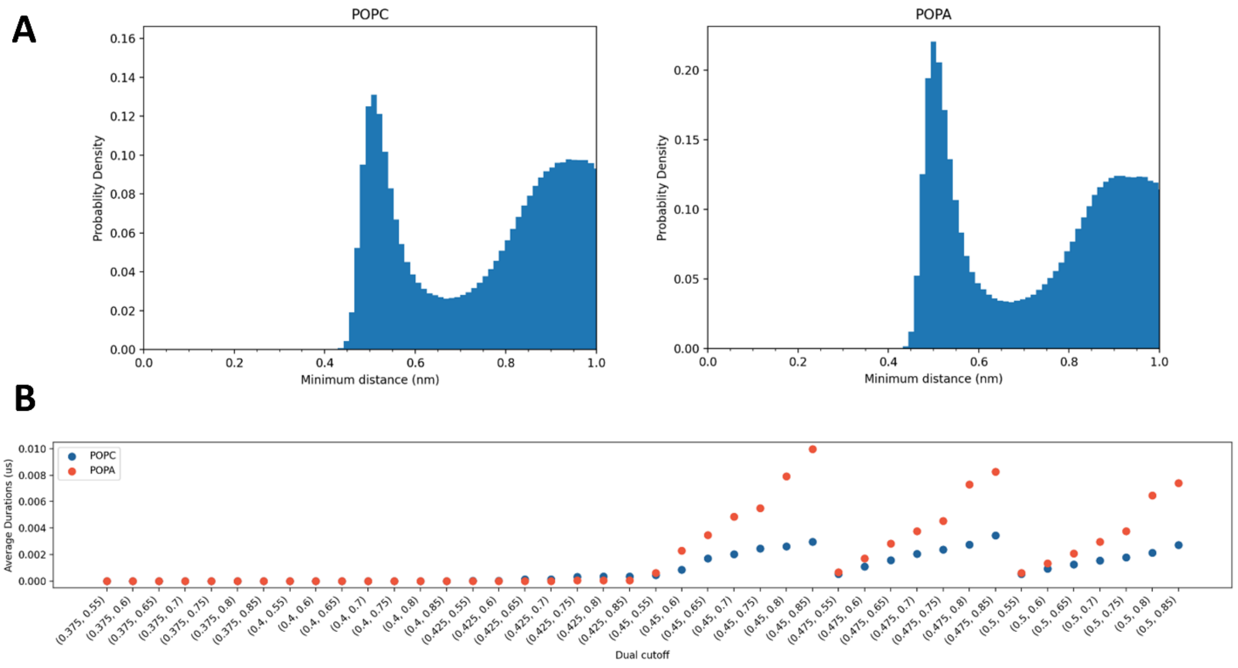

Figure S14. Cutoff determination for PyLipID calculations. A) Probability density of interactions between lipid headgroup bead and residue sidechain beads. Interactions taken for one simulation repeat using all headgroup-residue interactions exceeding 10 simulation frames. Left: data for POPC lipids. Right: data for POPA lipids shown for comparison. **B)** Average interaction durations calculated from tested cutoff pairs. The pair (0.5, 0.85) was selected due to being able to sample long interaction durations while sampling only specific lipid interactions.

Supplementary Note 1

CG-MD simulations

A different initial membrane configuration with randomized lipid positions was used for each of the three independent CG-MD simulation repeats. While these simulations are of sufficient length (30 μs) to sample of lipid binding to the nAChR, several analyses suggest that the dynamic binding reflects an "equilibrated" state:

First, despite the differing initial membrane configurations, each simulation repeat leads to essentially identical top-down 2D lipid headgroup density plots (Fig. S15) showing the same reported sites of lipid binding appear regardless of the starting membrane configuration. The data suggest that the 30 μs trajectory is sufficient for the lipids to sample the entire surface of the nAChR TMD.

Second, all percent occupancy/average maximum interaction duration plots (Figs. 4, 5, S4, S6, S7, S8 and S11) that were used to characterize lipid binding contain data calculated independently from each simulation repeat, with the data from each repeat averaged and plotted with standard deviations. The small standard deviations observed in the occupancy/duration plots show that similar dynamic lipid binding is captured in each simulation repeat.

Finally, an examination of the distribution of Chol over the course of a single 30 μs CG-MD simulation trajectory (Fig. S10) shows that despite Chol moving from the outer to the inner leaflet, and vice versa, the number of Chol molecules in each leaflet remains constant.

Atomistic Simulations

In atomistic simulations, equilibration of lipid binding sites is shown by both the swarm images and distance measurements between the lipid phosphate and its coordinating residues (Figs. 7 and S3). Specifically, lipids bind stably over the course of the 250 ns simulations, albeit with the precise lipid binding pose varying according to lipid motions (see swarm plots, Fig. 7b and S3). In addition, the phosphate of PA alternates between forming a salt bridge with either the +M3 arginine or -M4 positively charged residue throughout the 250 ns simulation. Finally, the pattern and distribution of PC and Chol binding to the nAChR is shown to be consistent at three separate time points (0, 125 and 250 ns) (Fig. S9).

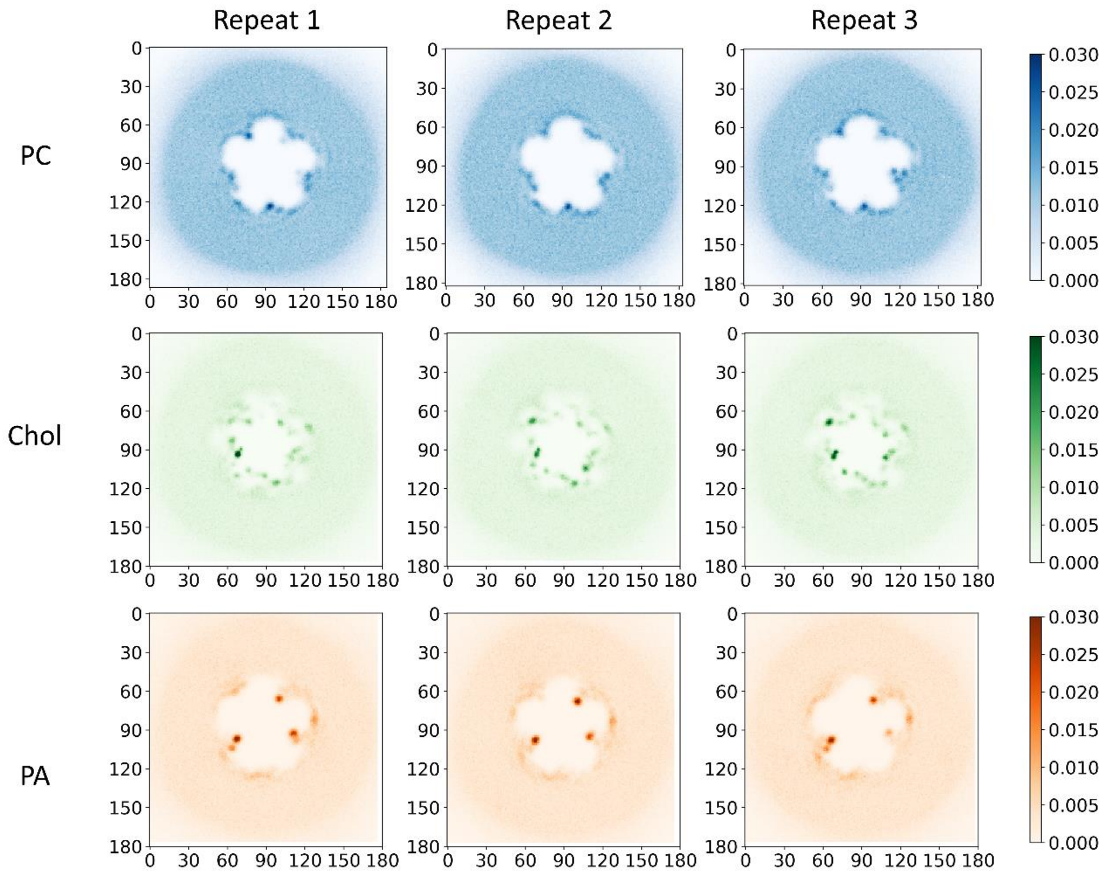

Figure S15. Top-down 2D lipid headgroup density plots for each of the three independent CG-MD simulation repeats. Density calculated over the course of each 30µs CG-MD simulation. Density is based on the "PO4" bead for both PA and PC, and the "ROH" bead for cholesterol. Results are shown for simulations of the apo state imbedded in 3:1:1 PC:PA:Chol.
