# Supplementary material for: State-dependent binding of cholesterol and an anionic lipid to the muscle-type Torpedo nicotinic acetylcholine receptor
Source: Commun Biol. 2024 Apr 10;7:437. doi: 10.1038/s42003-024-06106-8 (PMC11006840; doi:10.1038/s42003-024-06106-8)
Supplement: Supplementary file 3 — Description of Additional Supplementary Files [file 42003_2024_6106_MOESM3_ESM.pdf]

Description of Additional Supplementary Files

File name: Supplementary Movie 1

Description: Movie starts at 2.6 μs into a CG simulation repeat of the nAChR apo state in a 3:2 PC:Chol membrane and continues to 30 μs. Each frame duration is 2 ns with a frame rate of 60 fps. Chol is shown as green VDW beads with the bead corresponding to the hydroxyl group in red. The α and γ subunits are shown in pastel cyan and pink respectively, with the S235 bead shown in darker magenta. Subunits are shown as VDW beads with bonds as sticks

File name: Supplementary Movie 2

Description: The movie shows a 250 ns simulation of the apo nAChR in a 3:2 PC:Chol membrane. Each frame duration is 0.1 ns with a frame rate of 60 frames per second. Chol is shown as green sticks with the hydroxyl oxygen shown in red. The β and α subunits are shown as yellow and cyan cartoon respectively, with residues within 5 Å of the Chol molecules appearing as sticks.

File name: Supplementary Data 1

Description: The source data behind the graphs in the paper. Each text file contains occupancy or maximum interaction duration information for each residue of the nAChR. The text files are named as follows; for maximum duration: maxdur_[state]_[lipid]_in_[membrane composition]_[subunit]. For occupancy: maxdur_[state]_[lipid]_in_[membrane composition]_[subunit]. POPD is the lipid code for monoanionic POPA. Files ending in – sterol represent data for sterol group interactions, otherwise all data is for headgroup beads. Data from individual repeats is included in the last 3 columns of the text file, where for example "r1_occ" refers to occupancy data from the first repeat. The "Renumbered" residue column refers to physiological numbering, while the "Residue" column refers to CG numbering.
